# High Output Cardiac Failure Resolving after Repair of AV Fistula in a Six-Month-Old

**DOI:** 10.1155/2016/8564081

**Published:** 2016-01-14

**Authors:** Uygar Teomete, Rubee Anne Gugol, Holly Neville, Ozgur Dandin, Ming-Lon Young

**Affiliations:** ^1^University of Miami Miller School of Medicine, Department of Radiology, Miami, FL 33136, USA; ^2^University of Miami Miller School of Medicine, Department of Pediatrics, Miami, FL 33136, USA; ^3^Division of Pediatric Surgery, DeWitt Daughtry Family Department of Surgery, University of Miami Miller School of Medicine, Miami, FL 33136, USA; ^4^University of Miami Miller School of Medicine, Department of Surgery, Ryder Trauma Center, Miami, FL 33136, USA

## Abstract

*Background*. Acquired AVF in pediatrics are commonly caused by iatrogenic means, including arterial or venous punctures. These fistulae can cause great hemodynamic stress on the heart as soon as they are created.* Case*. A six-month-old 25-week gestation infant was referred for respiratory distress. Initial exam revealed tachypnea, tachycardia, and hypertension. There was a bruit noted on her left arm. An ultrasound showed an arteriovenous fistula. Its location, however, precluded intervention because of the high risk for limb-loss. An echocardiogram showed evidence of pulmonary hypertension that was treated with sildenafil and furosemide. However, no improvement was seen. On temporary manual occlusion of the fistula, the patient was noted to have increased her blood pressure and decreased her heart rate, suggesting significant hemodynamic effect of the fistula. The fistula was subsequently ligated and the patient clinically and echocardiographically improved.* Conclusion*. A patient in high output cardiac failure or pulmonary artery hypertension, especially prematüre patients with preexisting lung disease, should be probed for history of multiple punctures, trauma, or surgery and should have prompt evaluation for AVF. If it can be diagnosed and repaired, most of the cases have been shown to decrease the stress on the heart and reverse the pathologic hemodynamics.

## 1. Introduction

Arteriovenous fistulae (AVF) are anomalous communications between an artery and a vein. These malformations are known to increase cardiac output and have hemodynamic consequences [1]. They can be congenital or acquired. In the pediatric population, the most common cause of an acquired AVF is single or repeated diagnostic or therapeutic arterial or venous punctures. Particularly vulnerable patients include those with multiple medical problems, because of the need for chronic vascular access.

## 2. Case Presentation

**Figure 1:**
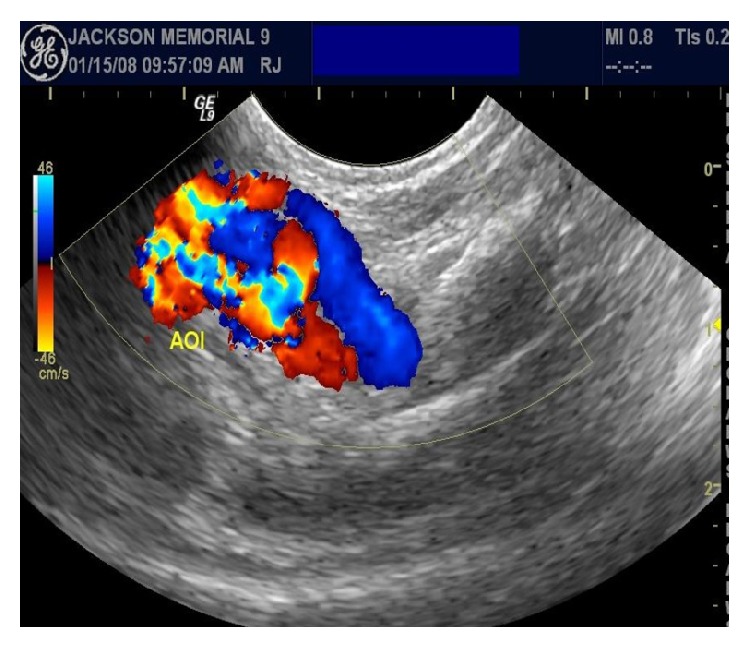
The distal left brachial artery to brachial vein fistula.

**Figure 2:**
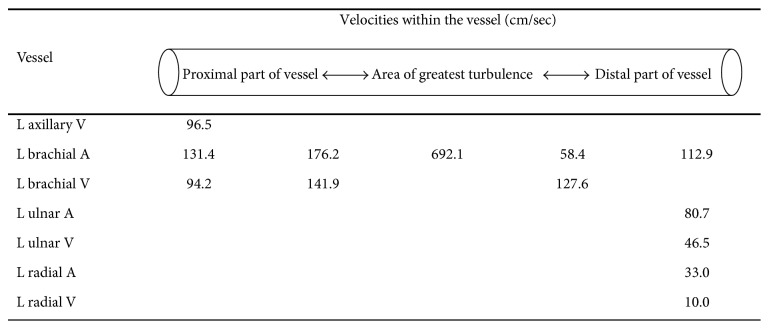
A representation of findings from the Doppler ultrasound of the left arm (L: left, V: vein, and A: artery).

A six-month-old female infant was referred to cardiology due to progressively increasing oxygen requirements and cardiomegaly on chest X-ray. She was born at 25 weeks of gestation and had multiple medical problems in the early neonatal period including retinopathy of prematurity, hyaline membrane disease, gastroesophageal reflux, severe liver dysfunction, and suspected necrotizing enterocolitis. She had a history of ligation of her patent arterial duct on the 16th day of life. On physical exam her respiratory rate was 65, her heart rate was 153, and blood pressure was 130/80 mmHg. Her oxygen saturation was in the low 90s on 30% FiO_2_ (fraction of inspired oxygen). Her lung sounds were coarse. Her heart had regular rate and rhythm, with no murmurs. Her abdomen showed a liver edge that was palpable three centimeters below the right subcostal margin. She had a palpable bruit at the left antecubital fossa. Pulses were normal and equal on all extremities, and there were no obvious discrepancies in limb lengths. An echocardiogram showed severe pulmonary artery hypertension with mild right ventricular and atrial dilatation, with a patent foramen ovale shunting right to left. Biventricular systolic function was normal, with no evidence of left ventricular hypertrophy or dilatation. A duplex ultrasound of the left upper extremity showed presence of an AVF between the left brachial artery and vein as evidenced by increased flow obtained from the left brachial artery with dilated, tortuous brachial veins demonstrating arterialized flow along the length of the veins. Velocity measurements of up to 692 cm/second were obtained at the level of the AVF (Figures 1 and 2).

The calculated blood flow in the fistula was estimated to be about 450–500 mL/min, which is equivalent to up to twice the estimated total cardiac output for this patient. The fistula was not ligated because its location was high risk for limb-loss. She was managed with diuresis and blood pressure control. One month later, she was reevaluated for recurrent and progressive episodes of desaturations and respiratory distress. On physical exam her vital signs showed a respiratory rate of 60 per minute, and oxygen saturations were between 90–95% on oxygen supplementation by nasal cannula. Her blood pressure was 52/32 mmHg, and the heart rate was 134–148 beats per minute. Cardiac exam demonstrated normal S1 and S2, no murmurs, and an intermittent gallop. Electrocardiogram showed normal sinus rhythm with right atrial enlargement and right axis deviation and T-wave inversion in the lateral leads, suggesting right ventricular strain. The echocardiogram suggested elevated pulmonary artery systolic pressures, which were 2/3 of the systemic pressure. The right ventricle showed mildly depressed systolic function.

The patient was started on sildenafil for management of the pulmonary hypertension, but this did not alter the measured pulmonary pressures upon echocardiographic reevaluation one week later. On careful assessment of the left antecubital fossa, with temporary manual occlusion of the affected area, there was a decrease in the heart rate from 128 bpm to 102 bpm, and an increase in the blood pressure from 76/55 to 92/57 mmHg (positive Nicoladoni-Branham sign [2, 3]). An echocardiogram that was done simultaneously showed a decrease in the flow through the right ventricle. The fistula was subsequently surgically ligated without complications. Repeat physical exam showed normalization of the patient's blood pressure and heart rate readings. She was quickly weaned to room air. A repeat echocardiogram done thirteen days after the repair showed no evidence of pulmonary artery hypertension.

## 3. Discussion

Arteriovenous fistulae are among the rare but well-documented causes of congestive heart failure [4–7]. In pediatrics, AVF can be congenital or acquired, the latter, not uncommonly secondary to iatrogenic causes including single or multiple arterial and/or venous punctures. The hemodynamic effects of AVF have been evaluated in many studies on patients in whom AVF are surgically created for hemodialysis [8, 9]. Cardiac output is said to increase greatly and immediately on opening of an AVF. This is secondary to an increase in the sympathetic tone, leading to increased stroke volume and heart rate. In only three days there are altered echocardiographic parameters evident after creation of an AVF. There have been notable increases in left atrial diameter, left ventricular diastolic dimension, shortening fraction, and cardiac output. There also are changes that suggest increased cardiac volume loading and decreased left ventricular compliance (decreased diastolic function).

There can be up to a 6-fold increase in blood flow in a single fistula [10]. “Fistular cardiopathy” is described in a study by Dallo et al. in 1984, when they studied 33 cases of acquired systemic arteriovenous fistulae from 1945 to 1981 and they observed that a syndrome of hyperkinetic hemodynamics resulting in heart failure developed from four days to 31 years after the initial insult and was related to the magnitude of the arteriovenous shunt [11]. The study by Iwashima in 2002 [8] showed that there is alteration in left ventricular diastolic filing pattern, which is suggestive of diastolic dysfunction. There is pseudonormalization of the systolic function as the left ventricular diastolic dimension increases with increased cardiac output. The left atrial systolic dimensions increased significantly within two weeks after creation of the fistula; left ventricular end diastolic dimensions increased significantly within one week. The left ventricular systolic dimension was not noted to change.

The above echocardiographic findings become more prominent as the heart is exposed to continued volume overload. Pulmonary artery hypertension, a less recognized consequence of AVF, has been described in many case reports. Peripheral arteriovenous shunting has been compared to the development of pulmonary hypertension due to congenital heart left-to-right intracardiac shunts [4]. Compensatory mechanisms from sustained volume overload and high cardiac output, over time, can lead to irreversible cardiac hypertrophy and ventricular dilatation. Pulmonary artery remodeling, consisting of endothelial proliferation, vascular smooth muscle hypertrophy, plexiform lesions, and other histopathologic changes, is seen in patients with left-to-right shunts (i.e., atrial septal defects, ventricular septal defects, or patent arterial ducts) which are all secondary to exposure of the pulmonary vascular bed to high volume [4, 6].

Pulmonary artery hypertension in our patient was complicated albeit masked by the presence of hyaline membrane disease from her prematurity, and the alterations in the right ventricular dimensions and function may already have begun earlier. The rapid progression of systolic dysfunction, however, shows the impact of the excessive venous return to the heart.

A small fraction of AVF spontaneously regress (<3%) [12]. Most of these lesions, however, need to be repaired. There have been cases wherein prolonged exposure to such a high output state predisposes the patient to even worse pulmonary artery hypertension after repair [13]. This underscores the need for urgent intervention in this lesion. In our patient the immediate effect of manually occluding the AVF showed a significant drop in the heart rate and an increase in the blood pressure, classically described as the Nicoladoni-Branham sign [2, 3].

The diagnosis AVF can be clinically made based on a simple palpable thrill and a machinery murmur over the affected area [14]. Other findings may consist of high cardiac output state, such as the Nicoladoni-Branham sign, peripheral edema distal to the fistula, limb length discrepancy, ulcers, or gangrene, related to insufficiency [15]. Diagnosis can be confirmed with color Doppler ultrasonography, digital subtraction angiography, or magnetic resonance angiography. The gold standard is to demonstrate on contrast angiography direct imaging of the abnormal arteriovenous communication and definition of the adjacent vessels [5, 16]. However systemic and neurologic complications related to contrast angiography occur in patients. Also hemodynamic and cardiac electrophysiologic changes are seen during iv contrast agent injection. MRI has the advantage of avoiding X-ray radiation exposure especially in pediatric patients. But patients with any metallic materials within the body and who have any history of claustrophobia are not suitable for this procedure. Color Doppler ultrasonography has high accuracy rates and can be performed fast and at bedside but usually it has operator dependent results. Additional to these diagnostic procedures, contrast enhanced ultrasound (CEUS) can be another option. AVF has risk of life threatening complications including spontaneous bleeding [17]. Compared to standard US, CEUS may be useful and has high sensitivity for detecting potential bleeding and active bleeding with low complication rates [18, 19]. When heart failure is present, electrocardiograms may show ST-T-wave changes from right and/or left ventricular strain, ventricular hypertrophy, and/or atrial hypertrophy [20]. Echocardiograms may show a hyperdynamic myocardium, volume overload, pulmonary hypertension, atrial and/or ventricular dilatation, and signs of possible diastolic or systolic dysfunction [8, 9].

In conclusion, acquired AVF in pediatrics are commonly caused by iatrogenic means, including arterial or venous punctures. These fistulae can cause great hemodynamic stress on the heart as soon as they are created. A patient in high output cardiac failure or pulmonary artery hypertension, especially in patient who is premature with preexisting lung disease, should be probed for history of multiple punctures, trauma, or surgery and should have prompt evaluation for AVF. If discovered, it should be addressed in a timely manner. If repaired, most of these cases have been shown to decrease the stress on the heart and reverse the pathologic hemodynamics.

## References

[1] Aitken E., Kerr D., Geddes C., Berry C., Kingsmore D. (2015). Cardiovascular changes occurring with occlusion of a mature arteriovenous fistula. *The Journal of Vascular Access*.

[2] Branham H. H. (1890). Aneurysmal varix of the femoral artery and vein following a gunshot wound. *International Journal of Surgery*.

[3] Velez-Roa S., Neubauer J., Wissing M. (2004). Acute arterio-venous fistula occlusion decreases sympathetic activity and improves baroreflex control in kidney transplanted patients. *Nephrology Dialysis Transplantation*.

[4] Bhatia S., Morrison J., Bower T., McGoon M. (2003). Pulmonary hypertension and arteriovenous fistulas. *Mayo Clinic Proceedings*.

[5] Pagel A., Bass A., Strauss S., Peleg E., Rapoport M. J. (2003). High output cardiac failure due to iatrogenic A-V fistula in scar: a report of a case and review of the literature. *Cardiovascular Surgery*.

[6] Gerke A. K., Wilson J. (2007). Complete resolution of severe high output heart failure and pulmonary hypertension after repair of longstanding arteriovenous fistula. *CHEST Journal*.

[7] Singh S., Elramah M., Allana S. S. (2015). A case series of real-time hemodynamic assessment of high output heart failure as a complication of arteriovenous access in dialysis patients. *Seminars in Dialysis*.

[8] Iwashima Y., Horio T., Takami Y. (2002). Effects of the creation of arteriovenous fistula for hemodialysis on cardiac function and natriuretic peptide levels in CRF. *American Journal of Kidney Diseases*.

[9] Basile C., Lomonte C., Vernaglione L., Casucci F., Antonelli M., Losurdo N. (2008). The relationship between the flow of arteriovenous fistula and cardiac output in haemodialysis patients. *Nephrology Dialysis Transplantation*.

[10] London G. M., Guerin A. P., Marchais S. J. (1999). Hemodynamic overload in end-stage renal disease patients. *Seminars in Dialysis*.

[11] Dallo L., Pastrana C., Rodríguez G., Medina Mora O., Barragán R., Bialostozky D. (1984). Acquired systemic arteriovenous fistulas: experience of 33 cases. *Archivos del Instituto de Cardiologia de Mexico*.

[12] Shumaker H. B., Wayson E. E. (1950). Spontaneous care of aneurysms and arteriovenous fistulas with some notes on intravascular thrombosis. *The American Journal of Surgery*.

[13] Nara T., Yoshikawa D., Saito S., Kadoi Y., Morita T., Goto F. (2001). Perioperative management of biventricular failure after closure of a long-standing massive arteriovenous fistula. *Canadian Journal of Anesthesia*.

[14] Cil B. E., Akmangit I., Peyniercioglu B., Karcaaltincaba M., Cekirge S. (2006). Iatrogenic femoral arteriovenous fistula: endovascular treatment with covered stent implantation and 4-year follow-up. *Diagnostic and Interventional Radiology*.

[15] Megremis S. D., Christaki M. A., Mourkoyiannis N. K., Papadopoulos G. S., Tsilimigaki A. M. (2006). Iatrogenic brachial arteriovenous fistula in a child. *Journal of Ultrasound in Medicine*.

[16] Das B. B., Sharma J. (2002). Acquired brachial arteriovenous fistula in an ex-premature infant. *Clinical Pediatrics*.

[17] Zorn K. C., Starks C. L., Gofrit O. N., Orvieto M. A., Shalhav A. L. (2007). Embolization of renal-artery pseudoaneurysm after laparoscopic partial nephrectomy for angiomyolipoma: case report and literature review. *Journal of Endourology*.

[18] Helck A., Hoffmann R. T., Sommer W. H. (2010). Diagnosis, therapy monitoring and follow up of renal artery pseudoaneurysm with contrast-enhanced ultrasound in three cases. *Clinical Hemorheology and Microcirculation*.

[19] Helck A., Sommer W. H., Wessely M., Notohamiprodjo M., Reiser M., Clevert D. A. (2011). Benefit of contrast enhanced ultrasound for detection of ischaemic lesions and arterio venous fistulas in renal transplants—a feasibility study. *Clinical Hemorheology and Microcirculation*.

[20] Ellis J., Martin R., Wilde P., Tometzki A., Senkungu J., Nansera D. (2007). Echocardiographic, chest X-ray and electrocardiogram findings in children presenting with heart failure to a Ugandan paediatric ward. *Tropical Doctor*.

